# The burden and impact of recurrent abdominal pain – exploring the understanding and perception of children and their parents

**DOI:** 10.1080/21642850.2022.2121710

**Published:** 2022-09-21

**Authors:** Sam Bradshaw, Aoife Brinkley, Barry Scanlan, Louise Hopper

**Affiliations:** a School of Psychology, Dublin City University, Dublin, Ireland; b Children’s Health Ireland (CHI) at Connolly, Dublin, Ireland

**Keywords:** Children, recurrent abdominal pain, family, quality of life, pain

## Abstract

**Background:** Recurrent abdominal pain (RAP) is a common complaint for children and can result in a significantly lower quality of life due to the extent it can interfere with normal life. RAP can also significantly impact the quality of life of parents. This study sought to qualitatively explore parents’ and children’s understanding and perceptions of the burden and impact of RAP.

**Methods:** Semi-structured interviews were conducted with a sample of parent/child dyads or families (*N* = 5) engaging with a psychology service.

**Findings:** The findings of the inductive thematic analysis revealed four emergent themes common to both parents and children: (1) Perception, understanding and identification of RAP, (2) Contributing factors, (3) Coping mechanisms/pain management strategies, and (4) Impact and burden of RAP.

**Conclusions:** These findings have important clinical implications regarding the identification and management of RAP and may also contribute to improving communication between clinicians, parents and children by providing insight from multiple perspectives into how RAP is experienced.

## Introduction

Abdominal pain is considered as a common complaint in children (Van Der Veek et al., 2013). For many this pain is only experienced on occasion, however for some, the pain does not resolve and can become chronic. This is commonly referred to as recurrent abdominal pain (RAP). RAP in children has been defined as a minimum of three episodes of pain that occur over at least a three-month period and affect the child’s ability to perform normal activities, which can have a significant impact on the child and their families (Chiou & Nurko, 2010; Reust & Williams, 2018). RAP is predominately considered as functional abdominal pain, in so far as no organic cause can be found on physical examination or investigation (Huertas-Ceballos et al., 2008; Rutten et al., 2015). Therefore, RAP can be a difficult diagnosis for children, parents and clinicians as there is no definitive cause of the symptoms (Van Tilburg et al., 2006). As such, it is important that clinicians communicate effectively with children and their parents. A better understanding of the perspectives of children and families can enhance this communication. This study aims to explore parent’s and children’s experiences and perspectives of RAP and its potential causes, impacts and burden in order to gain a greater overall understanding. This information will help inform clinician’s understanding of how children and parents conceptualise RAP and how the role of psychological factors can be explained to them. This insight will better guide treatment for children with RAP and thus improve outcomes such as well-being and quality of life and thereby reduce the impact on families.

Children affected by RAP report significantly lower quality of life than their peers (Chiou & Nurko, 2010; Korterink et al., 2015; Warschburger et al., 2013). The severity of symptoms can affect a child’s quality of life in various ways (Youssef et al., 2006) usually as a result of the frequent interference with daily life, school absenteeism, withdrawal from activities and disruptions in peer relationships (Sjölund et al., 2020; Varni et al., 2015). RAP can often give rise to prolonged, unnecessary diagnostic testing and protracted attempts at treatment, which can manifest in increased anxiety and a further reduction in quality of life for the child and their parents (Bufler et al., 2011; Calvano & Warschburger, 2018). If left untreated, there is a considerable chance RAP will remain into adulthood, lead to other psychopathology, or into additional somatic symptoms (Gieteling et al., 2008; Newlove-Delgado et al., 2017; Youssef et al., 2008).

RAP is associated with extensive diagnostic investigation, thus placing a significant burden on the healthcare system (Gieteling et al., 2011; Sjölund et al., 2020). RAP can also cause a significant degree of anxiety and worry for parents/care givers as they commonly feel helpless in their inability to relieve the pain while also still fearing a serious organic root (Bremner & Sandhu, 2009). As a result, families of children with RAP generally have poorer family function than healthy populations due to the negative impacts on family life (Brodwall et al., 2018).

A direct cause of RAP is often difficult to establish and therefore successful management can prove complicated. However, efforts are on-going to identify the causal and contributing factors to RAP, with Engel’s (1979) biopsychosocial model often proving to be a useful method of conceptualising its aetiology (Hyams & Hyman, 1998; Rutten et al., 2015). It is suggested that physical, emotional, and environmental factors may contribute to the manifestation of unexplained abdominal pain (Newlove-Delgado et al., 2017). Brown and colleagues (2016) suggest that an inter-play occurs between the factors surrounding the child, including genetic predisposition, life events, the family and the child’s coping mechanisms for dealing with stress and pain. It is also considered that RAP is strongly associated with stress and psychological disorders such as anxiety and depression, thus psychosocial interventions are often recommended (Friesen et al., 2021; Rutten et al., 2015). Indeed, these psychological factors are thought to play a significant role in the development or exacerbation of abdominal pain, with the neural pathways along the ‘brain-gut axis’ being postulated as laying the physiological foundation for the integration of psychological experiences with abdominal pain (Masters, 2006; Plunkett & Beattie, 2005). Stressful and traumatic life events such as the disruption of personal relationships are also believed to be potential contributors for RAP (Boey & Goh, 2001; Robinson et al., 1990; Sonneveld et al., 2013).

Once no organic disease has been established and a diagnosis of RAP has been made, individuals are usually managed by reassurance and simple measures such as distraction techniques, whilst a wide range of psychosocial interventions are recommended in more difficult cases (Brown et al., 2016; Huertas-Ceballos et al., 2008; Plunkett & Beattie, 2005). However, it is important to note that no specific ‘organic’ cause or biological contributor identified is not to suggest that there is no disease, or that symptoms are not real or without any biological basis. Indeed, when no specific disease entity has been identified as a cause for the child’s symptoms it does not necessarily mean that there are no biological factors involved that may be in need of addressing through gastrointestinal medications or other biological treatments, alongside psychosocial intervention (Friesen et al., 2021). The psychosocial interventions for treating RAP can include cognitive behavioural therapy (CBT), hypnotherapy, written self-disclosure, yoga and online guided therapy treatments. However, there does not appear to be consensus amongst clinicians on what treatment approach should be offered to patients. As such, treatment of RAP remains inconsistent due to the lack of confirming evidence for any one approach (Abbott et al., 2018; Quak, 2015; Weydert et al., 2003).

In order to develop effective treatments, it is imperative to have a complete understanding of the different factors that cause and maintain RAP (Van Der Veek et al., 2012). Parental influence is a significant factor, as parental attitudes and responses to pain may be important for helping children with their RAP (Brodwall et al., 2018; Simons et al., 2008; Van Der Veek et al., 2012). Parental attributions for their child’s pain are thought to be influential in affecting the course of symptoms (Ramchandani et al., 2011). Indeed, a previous Irish study found that children whose parents held psychological attributions such as stress-related illness for their RAP had better outcomes than those who rejected a biopsychosocial model of illness (Crushell et al., 2003). Furthermore, there seems to be a consensus across the literature that the early introduction of a biopsychosocial model of illness with both the parent and the child can improve outcomes considerably (Brown et al., 2016; Schurman & Friesen, 2010).

There is a body of literature documenting the association between parental behaviour and children’s pain, with such studies identifying that children with chronic pain are more likely to have parents with chronic pain, and that parents with chronic pain report increased pain sensitivity in their children (Evans et al., 2010). A review of this specific literature found important causal factors included the parent’s psychological history as well as processes related to family functioning (Evans et al., 2008). Indeed, as parents are the primary models for children, parents who experience physical symptoms themselves may model a focus on physical symptoms to their children (Van Der Veek et al., 2012). Furthermore, children will be more likely to adopt modelled behaviour if it results in positive outcomes rather than if it has unrewarding or negative effects (Bandura & Walters, 1977; Ramchandani et al., 2011; Van Der Veek et al., 2012). Therefore, if the child is allowed to stay home from school/come home early and watch television or play video games, this parental behavioural response may maintain or exacerbate the child’s pain behaviour (Van Der Veek et al., 2012).

Although some studies have looked at parents’ experience in relation to RAP (Brodwall et al., 2018), there has been very little research looking at the perspective of children who experience RAP in terms of causes, impact and coping (Ramchandani et al., 2011). Understanding how children experience the processes of causation, prevention and treatment of their illness and pain, is necessary to help health professionals in their work with children (Koopman et al., 2004). Thus, an improved understanding of the child’s experience and perspective will better enable clinicians to develop suitable interventions.

Factors that might enhance clinicians’ communication with children and their parents has only relatively recently emerged as an issue worthy of research (Carter, 2002; Levetown, 2008). In addition, there is a dearth of research on children and parents perspectives on the causes of RAP and on their view of the burden involved for children and their families. Indeed, children’s voices in particular, as well as those of their families do not figure significantly in the healthcare literature. The lack of information on how children and parents understand RAP is likely to impact clinician’s ability to communicate effectively. While psychological therapy is at the cornerstone of treating children with RAP, the role of emotional factors such as anxiety can be a difficult concept to understand. As a result, it can be challenging for clinicians to communicate the role of such factors to patients and children. A better understanding of the perspectives of children and families can enhance this communication.

This study aims to explore parents’ and children’s experiences and perspectives of RAP and its potential causes, impacts and burden. To the researcher’s knowledge, no study has previously investigated this area using a qualitative methodology that includes both children and their parents. It is therefore hoped that a greater understanding will be gained in terms of each individual’s perception of RAP as well as their experiences of living with it. This information will help inform clinician’s understanding of how children and parents conceptualise RAP and how the role of psychological factors can be explained to them. This insight will better guide treatment for children with RAP and improve outcomes such as well-being and quality of life. Reducing the impact of RAP on children and families will enable them to go about their daily lives more easily and this is beneficial to the community as a whole. As such, this is an important area of research in terms of understanding the experience of RAP and how to positively address its impacts on both children and their parents.

## Materials and methods

### Design

This research study employed an exploratory qualitative design. The study involved one-to-one, audio-recorded, semi-structured interviews with parent and child independently.

### Participants and recruitment

Participant parent/child dyads or families (*n* = 5) were recruited through the psychology department, within the general paediatric outpatient service of an Irish hospital. All participants were attending the psychology service at the time of interview. In order to participate in this research, children needed to be aged between 9 and 16 years, have a diagnosis of RAP and be experiencing symptoms. In total, 11 participants took part in the study; five children (two males and three females) ranging in age from 9 to 16 years; and six parents (one male and five females).

Verbal and written information on the study was provided to parents by a member of the psychology team during a regular psychology appointment. Parents interested in volunteering could make contact directly with the researcher or could respond via their psychologist and an interview was arranged.

### Materials

Interviews were conducted online via a video conferencing application, due to the concurrent Coronavirus pandemic. Interviews were semi-structured and questions were developed from a combination of those based on previous related research (Brodwall et al., 2018; Perrin & Gerrity, 1981; Smart & Cottrell, 2005; Von Baeyer, 2007) in collaboration with an expert in the diagnosis and treatment of RAP. Interviews began and ended with ‘problem free talk’ to help participants feel comfortable with the interview process (Ratner et al., 2012).

Topics for the interviews were geared at revealing the perceptions and individual experiences of RAP, views on potential causes and ways of coping, as well as the impact on quality of life. Interview questions differed for the child and parent groups and age appropriate language was used for the child interviews. The child interview questions primarily focused on their own experience of the pain, the causes and how they perceived them, managing it and how it impacts their lives. Parents were questioned on when their child’s pain commenced, the frequency and consistency of the pain and how it manifests and is managed. Questions also covered how their child’s stomach pain impacted family life. Parents were asked what they felt caused or contributed to the pain and how the pain was discussed within the family. Parents were also asked to describe how their child’s pain impacted their own quality of life.

### Procedure

Written consent was obtained for parents and children prior to interview and verbal consent was received before starting the interview. Participants were reminded that they could withdraw from the study at any point without it affecting their further treatment. Participants were also asked at the halfway stage if they were happy to continue.

Children and parents were interviewed separately, unless the child was not comfortable to do so, in which case the parent was present. In these cases, parents were informed that they should refrain from taking part, interrupting, or answering questions on their child’s behalf. Four of the five children requested that their parent remained present during their interview. Child interviews ran from between 15 and 20 min while parent interviews ran from between 20 and 40 min approximately. All interviews were audio-recorded. A pilot study was conducted (one adult and one child) prior to full data collection (this was not included in the final analysis). Data were transcribed by the researcher and then analysed using Inductive Thematic Analysis (Braun & Clarke, 2006).

### Ethics statement

This research study received ethical approval from the research and ethics committees in the respective university and hospital. Consent was obtained for all participants included in this study.

## Findings

A total of four themes emerged from the dataset. The themes were the same across the parent and child participants, however, differences emerged at the subtheme level. The themes and subthemes identified are displayed in Table 1.

**Table 1.** Emergent themes and subthemes.

Parent themes and subthemes	Child themes and subthemes
*Theme 1*: Perception, understanding and identification of RAP ** ***Subtheme 1*: Initial pain identification, manifestation and development ** ***Subtheme 2*: Discovery journey to understanding RAP	*Theme 1*: Perception, understanding and identification of RAP *Subtheme 1*: Perception of causes *Subtheme 2*: Pain identification, manifestation and development
*Theme 2*: Contributing factors ** ***Subtheme 1*: Internal factors ** ***Subtheme 2*: External factors	*Theme 2*: Contributing factors *Subtheme 1*: Internal factors *Subtheme 2*: External factors
*Theme 3*: Coping mechanisms/pain management strategies ** ***Subtheme 1*: Strategies/techniques ** ***Subtheme 2*: Awareness/familiarity to the pain over time ** ***Subtheme 3*: Role of psychology	*Theme 3*: Coping mechanisms/pain management strategies *Subtheme 1*: Strategies/techniques *Subtheme 2*: Support systems *Subtheme 3*: Child resilience and determination
*Theme 4*: Impact and burden of RAP ** ***Subtheme 1*: Emotional reaction and impact ** ***Subtheme 2*: Impact on sleep and school ** ***Subtheme 3*: Impact on child’s quality of life ** ***Subtheme 4*: Impact on parents and family quality of life	*Theme 4*: Impact and burden of RAP *Subtheme 1*: Emotional reaction and impact *Subtheme 2*: Impact on sleep and school *Subtheme 3*: Impact on quality of life ** **

Each theme is presented in the following sections; parents (P#) followed by children (C#). Anonymised direct quotations provide context and serve as anchor examples for each.

### Emergent themes: parents

#### Theme 1: Perception, understanding and identification of RAP

All parents highlighted that the initial stages of RAP brought about high levels of uncertainty and confusion. The pain and symptoms seemed to manifest and develop in different ways over time, making it difficult for them to identify potential causes or contributors. Parents also commented that the process of determining the cause of the pain, and the elimination of potential causes was lengthy.

##### Initial pain identification, manifestation and development

All parents talked about the fact that the problems experienced by their child were initially understood to be a potentially serious physical illness with multiple symptoms. The pain frequency and pain development made the process of obtaining a clear diagnosis more difficult.

she got a bug or something, an infection, a viral thing … she vomited for a week. We brought her to hospital, she vomited for another week, she stopped vomiting, she went back to school and started vomiting again, so we had a load of tests and things then and that’s when it started. (P2)

at the start I actually thought it was like gastric bug … I brought him to my GP … he thought maybe it was his appendix or stuff like that, but that was all ruled out  …  then we started to discover by ourselves that it has to be something got to do with anxiety. Ya know because there was no … his bloods were fine, everything was fine. (P6)

Parents spoke about how the pain appeared to manifest and develop in different ways.

It’s random. In the beginning it was quite often … initially like it was kind of like the same thing. But now it’s irregular. (P2)

it actually started more that she described it in her throat … like a blockage in her throat and it felt like she couldn’t eat and there was actually something physically in her throat. And then it kind of linked a bit later on to her tummy and sometimes it’s the two together. (P1)

##### Discovery journey to understanding RAP

Parents’ narratives all revealed a similar experience of RAP. They described a journey beginning with uncertainty of not knowing, going through the stages of ruling out medical conditions and trying to pinpoint the problem, leading to the role of psychology in identifying and generating an understanding of RAP.

initially we didn’t know what was happening. (P2)

we didn’t know at the start was he sick, was it just in his head … was it genuinely something upset in his tummy. (P6)

After alluding to this initial uncertainty, parents next discussed the process of ruling out serious illness and finding answers and a greater understanding of their child’s situation. They all mentioned how long and frustrating the process was.

they did all the tests, scans, MRIs all that kind of stuff just in case it was a tumour or anything bad, ehm it still wasn’t fixed … we were worried it was more serious before the tests, but once we had the tests then we were comfortable that it wasn’t anything more significant. (P2)

got him to the doctor … 18-24 months later we’ve got him in for … a full medical MOT … and all of that came that he was clear. And after that obviously there’s still something wrong. (P4)

Parents only spoke about engaging with psychology when serious medical issues had been ruled out. They felt that this played a role in helping to identify and gain a better understanding of RAP.

we did some sessions in the hospital … that’s kind of helped her to kind of identify what it is more, where she would have said it was a physical … now she’ll kind of say I think it’s a worry pain. I think I’m sad about something or I’m worried about something … so ya know we’re kind of getting to the root of it quicker I suppose, that she’s able to identify it. (P1)

#### Theme 2: Contributing factors

The theme of contributing factors to RAP featured heavily throughout the parent interviews. Parents’ narratives revealed two sets of contributing factors; internal and external and the two appeared intrinsically linked.

##### Internal factors

Anxiety and anticipatory worry, particularly for significant or large events, were internal factors identified with children’s experiencing of stomach pain by parents. Parents also reported their children to be sensitive and deep thinkers.

he’s a very sensitive young fella and I think that he does feel the effects of anxiety … and you know there’s no way he doesn’t get the impact of that. (P4)

In anticipation of something, maybe at school, or ya know, a concert, or something like that. Especially if there’s something bigger kind of out of the norm. (P1)

##### External factors

Social situations, school and bullying featured heavily in the data as likely stressors contributing to RAP and ‘bigger kind of flare-ups’ (P1). The role of school as a particular stressor was revealed to be especially salient as a result of the COVID-19 pandemic. This resulted in the children being home-schooled and not experiencing RAP and were also reported to have engaged more in their school work. Other external factors such as family issues, including an ill parent and seriously ill sibling also featured as contributing factors.

he used to get little pains … because he didn’t like the teacher in the school, she was very strict … I’d say there were pains once every week … and then he was off for the holidays or he was on school holidays, he never had a pain in his tummy. (P6)

at its worst a couple of years ago she was having a difficult time in school … it was in a sense of bullying … she’s quite a sporty person … and other girls were just, making like smart comments and stuff like that. (P5)

since the COVID, since he’s been at home with me … I’ve noticed there’s no pain … but when he was in school, yeah. (P6)

#### Theme 3: Coping mechanisms/pain management strategies

All parents described multiple coping mechanisms adopted by themselves and their children to deal with RAP. These varied throughout the parents’ narratives in perceived effectiveness and in how frequently they used them. Analysis of parental data revealed three subthemes that address the main theme.

##### Strategies/techniques

Parents alluded to a number of strategies they or their child would tend to initially use in order to help their child manage and cope with an episode of pain.

So she kind of has things that she can do to manage it … like the kind of the getting air, the drinking ginger tea, drinking water … she kind of has a handle on it. (P2)

when she gets the pains she feels like she has to be in a bathroom. (P5)

Reassurance and a calm approach were prominently cited by parents as being one of their initial techniques to dealing with an episode of pain.

I’d reassure him that everything is ok and that his tummy pain will go away, he just has to relax. (P6)

I try to just stay calm and talk through, see if we can figure out what it is or what might help. (P1)

Distraction techniques were also reported as approaches often adopted by parents in terms of managing pain. Breathing exercises and bedtime meditation were also cited as being effective.

we have that dog so that’s been brilliant … some outdoor time … he likes cooking, he likes baking, so anything that breaks him away … that allows him to talk. (P4)

Parents also described having an open dialogue with their child and actively communicating with them in efforts to find out what might be contributing to their pain as well as helping them to understand it.

just keep talking about it … an open dialogue, just tell her if she has any problems she can talk to us. That kind of thing. (P3)

##### Awareness/familiarity to the pain over time

Another form of coping mechanisms that emerged from the data was the idea of both parent and child developing a greater awareness and familiarity with RAP over time, and a new form of reassurance that developed through experience. This and other learned coping mechanisms were portrayed by parents as effective forms of pain management.

In the beginning it was very, very bad … now I suppose if it starts she kind of deals with it immediately so in the beginning she wouldn’t have had coping mechanisms for it and it could have got worse. (P2)

##### Role of psychology

All parents also made reference to the positive impact seeing a psychologist had on their child, and the coping strategies and greater understanding and awareness that had come from this process. Parents noted that having a safe place to talk and open up and improvements in how their child understands their RAP and expresses related emotions were beneficial.

that’s helped a lot actually … now I don’t know what the conversations were but she always came out in really good form, I always felt like when she was in there she was able to open up to the psychologist. And the pains kind of started reducing … like the bouts wouldn’t be as frequent, or haven’t been as frequent since she started going to the psychologist. (P5)

#### Theme 4: Impact and burden of RAP

All parents described the burden of RAP felt by their children. The impact of RAP also appeared to be significant on their children, their families and on themselves.

##### Emotional reaction and impact

All parents described their child experiencing some level of emotional distress, either as a reaction to pain or as a consequence of their situation.

they affected him quite bad actually … yeah they did he just cries … and won’t go anywhere, just stays put. (P6)

She gets kind of, I suppose a bit angsty. And ya know feels like she can’t, a bit stressed, like she can’t figure it out she can’t solve, it won’t go away. (P1)

##### Impact on sleep and school

Experiencing pains in school and the impact this can have on concentration and performance were also highlighted by parents. Attendance at school was also impacted, with some children going into school late, coming home early or missing periods of class as a result of their RAP. However, most parents stated that their child would not be entirely absent from school due to their RAP.

it takes a bit out of her, ya know, it can affect her sleep and that affects her. If she’s tired the next day, if she’s grumpy the next day, unhappy to go to school, ya know the day after. (P2)

I do remember the teacher saying it to me … he has the pain and he just can’t concentrate … he’s trying his best and the pain is taking over the mind. (P6)

he has never missed school, he’s always gone in and once or twice I was called to pick him up but that was it. (P4)

##### Impact on child’s quality of life

Parents also commented on the different ways RAP impaired their child’s quality of life. However, the majority of parents did not see it having a significant impact on their child’s friendships and social life when explicitly asked and did not express current concerns in these areas. When asked whether their child’s pain stops them from doing things one parent responded: ‘at the height of it, definitely yeah’ (P3).

there have been times he just couldn’t, you know … he didn’t want to do a birthday, or he didn’t want to go somewhere. (P4)

her reaction causes her not to enjoy it as much as she should or to make a bigger deal of it … I don’t think it stops her that often but it just makes life much more difficult by making a bigger deal out of it. (P1)

##### Impact on parents and family quality of life

Parents discussed the impact that the child’s RAP had on themselves and on family quality of life in general. The impact in the earlier stages of their child’s RAP appeared to be highly salient among parents’ responses. Parental concerns and worry in the early stages featured prominently among the parent interviews. In particular, parents expressed their initial concerns of not knowing what was wrong with their child and how it ‘was quite a worry in the beginning’ (P2).

At that time it impacted an awful lot yeah. It was very stressful for everybody, because we didn’t know at the start was he sick, was it just in his head. (P6)

Most parents disclosed that they were less concerned once their child had undergone medical tests, and serious illness had been ruled out. Some still had worries it could be something more serious. Indeed it was evident from the parent interviews that they too experience much stress and anxiety as a result of their child’s pain, impacting their quality of life and that of their whole family.

I’m probably going to push … just to even get a camera down to have a look and just make sure … I just want to make sure that I’ve done everything possible. (P5)

the odd time you kind of do have that thing of maybe it is actually you know it’s a physical pain or something that needs medical attention. (P1)

I suppose I would like her to not have this, just to be able to get on with her life and not have to worry about it. (P2)

### Emergent themes: children

#### Theme 1: Perception, understanding and identification of RAP

The children described their perceptions of the causes of stomach pains. The children also outlined how they conceptualised their symptoms of RAP and spoke about their understanding the RAP.

##### Perception of causes

Children’s perception of the causes of RAP included a combination of diet and worry or external factors.

when they eat something bad … or when they’re worried. (C3)

sometimes it’s when you’re nervous or eh when things change or stuff. (C1)

##### Pain identification, manifestation and development

It emerged from the data that each child had a somewhat unique experience of how they identified their pain and also how it manifests. Dual and multiple symptoms were identified by most of the child participants.

I get like pains in my throat or my belly … I used to just get it in my throat but then a while ago they started in my belly as well … It feels as if like there is a big lump stuck in my throat or in my belly or something. (C1)

Well sometimes I just get a headache or just tummy pains, and sometimes it’s nauseous and sometimes it’s more ‘ouch!’ (C2)

How the pain occurs, manifests and develops over time also appeared to be unique among the children.

Well I was in PE in school I was in first year and my stomach just got really acidy and I had to go to the bathroom and then we went to the doctors. (C4)

I’d just come back from school and I started feeling really, really sick and then I like, I got sick like a few times, like a tummy bug but then that kept happening for ages and ages and then after a few months I stopped vomiting and it just became headaches and tummy pains. (C2)

#### Theme 2: Contributing factors

The children described multiple internal and external factors and stressors that they believed contributed to their RAP. They are presented as two separate subthemes for clarity, although the two appear intrinsically linked.

##### Internal factors

Feeling sad or worried was explicitly identified with experiencing stomach pain by four of the five children. Feelings of nervousness and anticipatory worry were also highlighted as being associated with stomach pains.

I think it’s just because I’m nervous or something and my whole body feels it nearly … they kind of happen more if I’m worried, but sometimes when I’m sad as well. (C1)

like if I’m nervous for something … or if I have something coming up and I don’t want to do it or … eh I definitely get them when I’m worried but I don’t think they’re as bad as when I eat something. (C4)

Unlike the other children, C5 commented that: ‘I’d only have a pain if my mam wasn’t there’ (C5).

##### External factors

Children also frequently mentioned negative and unpleasant environmental factors as triggers for their pain.

when I get angry sometimes … ya know people when they say mean things to my friends and stuff, ya know sometimes it makes me very annoyed and not feel good so sometimes I get a headache or a tummy pain when I’m feeling stuff like that … I can’t deal with some people like I sometimes have to but like whenever I do I just always feel very sick. (C2)

Like I would be nervous about going to school or going anywhere like if I was just to go out to cycle my bike. (C4)

#### Theme 3: Coping mechanisms/pain management strategies

The children described their perceptions of pain management, methods of coping with RAP and managing their pain.

##### Strategies/techniques

The children referred to a wide range of strategies that help them to cope with episodes of pain. Generally, the children spoke of having a glass of water, getting fresh air, sitting down, taking some time out and trying to relax with enjoyable tasks or activities. They also talked about distracting themselves, for example, engaging in certain activities such as computer gaming and sports. These were identified as pain-free experiences by some of the children. Breathing exercises and techniques were also highlighted as a useful approach to calming down during a pain episode.

usually if I relaxed, then it would just stop … I usually take a bath or watch a movie with my mam and dad. (C5)

I started thinking about different things and then when I was thinking about different things the bad thoughts came out of my head so it wasn’t bothering me anymore. (C1)

when I’m training … that’s the only time that I don’t be like nervous about something  …  but anytime I go to go training, it just never happens. (C4)

##### Support systems

Having support from parents, other family members and friends also emerged as an important coping mechanism from the children’s narratives. A significant number of references were made to having an open dialogue with parents and family members and talking things through in relation to what they were feeling. Whilst the importance of having someone to talk to about one’s experience was conveyed in the data, getting reassurance also featured prominently.

I talk to them about it sometimes and then we can figure out a way of getting the worries out of my head. (C1)

But if they’re bad then I’d probably ask my mom to come upstairs and then I’d just tell her like, its worse this time and … she’d just tell me I’m fine … it’ll be ok like. (C4)

##### Child resilience and determination

Many of the children reported getting on with things, like school, sports, chores, and other tasks and activities despite experiencing pain. Some of the children appeared determined to not let the pain stop them from doing things, while it also appeared that a level of awareness and familiarity with their pain contributed to an overall resilience. As such it was considered that child resilience and determination were forms of coping mechanisms built up over time.

I know there are worse things obviously so I just like ya know grit and bare it. (C2)

I try to not let it stop me from doing anything, I try to just kind of get on with it, and like it’s going to happen anyway so I’m better off doing something or not. (C4)

#### Theme 4: Impact and burden of RAP

RAP emerged as a significant burden on the lives of the children included in this study and its impacts spanned a number of different areas, all of which could be seen as negatively impacting quality of life.

##### Emotional reaction and impact

All children conveyed some level of emotional reaction to experiencing RAP. Some reported being afraid, upset or feeling bad, while others became annoyed and frustrated, leading to upset. C2 highlighted the fact that RAP was having an emotional impact on them when it stopped them from doing things. One child acknowledged that when they first got the pains it made them ‘afraid and upset’ but ‘not really anymore’ (C5).

it’s really annoying because sometimes like, you could be looking forward to something and you wake up that morning and then your stomach is just like no … yeah annoyed and kind of like … why?  …  like today of all days. (C4)

I don’t like getting them and I wish I didn’t … it’s annoying when I can’t do things because of it and when I’m like ya know I feel really bad because of it … sometimes it does make me a bit grumpy because it is just I want to do all the things but ya know then I just can’t. (C2)

##### Impact on sleep and school

The impact on sleep was highlighted by four of the five child participants, with one stating problems with sleeping, but not attributing it to RAP. The impact that RAP had on sleep for the participants seemed to run in tandem with its impact on the school and school work, particularly effecting concentration. The impact of RAP on the school could also convey in how children talked about the way in which it made school more difficult and how they sometimes had to take time out from class.

sometimes it keeps me up really late … a lot of nights … it definitely makes it harder to sleep and sometimes it affects my dreams … because I can’t sleep at night ehm it’s really hard to do some of my work. (C1)

when there was school days it was quite annoying because I would always feel very sleepy. (C2)

like I could miss a bit of class if like I have to be out of class for 20 minutes or something. (C4)

##### Impact on quality of life

Children tended not to speak directly about the impact of RAP on their quality of life but this was perhaps implicitly conveyed through their references to the emotional impact of RAP, as well as the direct impact it can have on school and performance. In addition, the impact that RAP can have on a child’s social life and how it can act as a barrier to doing various things were highlighted by a number of participants in this study.

ehm … just often playing … and kick boxing. (C5)

like to go out or to be around people because then I just feel like, if my belly is sore outside with people or I’m not at home its really uncomfortable because I’m just like … you’re not at home or its just really uncomfortable … it would definitely make me think of not going. (C4)

In contrast, two participants (C2 and C3) stated that they would not normally get stomach pains when they were with their friends, with one responding; ‘not as often as I would other places but ya know I still do’ (C2). The impact of RAP on quality of life was further highlighted when participants were asked what difference they think it would make if they were not experiencing the stomach pains at all, with one child simply responding; ‘happier’ (C3).

a big, big difference … it would be like life changing cos then I could actually you know, do stuff. (C5)

I’d feel lot freer, cos I could do whatever, without having to worry about it. But then it’s always in the back of my head that it could still come at any time of the day, you don’t necessarily have to wake up with it. (C4)

## Discussion

The purpose of this research was to qualitatively explore parents’ and children’s experiences and perspectives of RAP, the potential causes and its impacts and burden, therefore gaining a greater overall understanding of this condition. The findings of the inductive thematic analysis revealed four emergent themes common to both parents and children; however, differences were evident between the two groups in terms of the composition of subthemes. The themes and subthemes identified will now be discussed.

### Theme 1: Perception, understanding and identification of RAP

It was apparent from the parental data that the initial stages of RAP brought about high levels of uncertainty and confusion. Many of the parents described the initial conceptualisation of a problem as a serious physical illness with multiple symptoms, many of which have been described in the existing literature (Abbott et al., 2018). The pain and symptoms were reported to manifest and develop in different ways over time, making identification difficult and leading to an oftentimes lengthy process of elimination before a clearer understanding and awareness is obtained. This journey of uncertainty and not knowing was something all the parents alluded to as they recalled trying to obtain a diagnosis. This prolonged process or journey of uncertainty that the parents described is congruent with the literature and is a common experience for parents of children with RAP (Bufler et al., 2011). It was evident from responses that this drawn-out process caused confusion and significant worry for the parents, which again, would appear to be a common occurrence for many families in this position (Calvano & Warschburger, 2018). Following diagnostic testing and a ruling out of any serious medical issues, parents generally felt more comfortable, but were aware that there was still something wrong. This typically led to engaging with psychology services. A key finding of this study was the important role parents attributed to psychology in helping them and their children identify and gain a better understanding of their child’s situation. Therefore, whilst ruling out serious medical concerns is important, a novel finding was that the role of psychology was integral to the process for the parents of gaining a greater understanding of RAP and helping to identify it and therefore reducing levels of uncertainty.

In contrast, each child had a somewhat unique experience of how they identified their pain and how it manifested for them. The children, similar to their parents, reported multiple symptoms occurring with their RAP as established in the existing literature (Huertas-Ceballos et al., 2008) but also alluded to the changes in its development over time, which to the researcher’s knowledge is a novel concept. Some of the children recalled their initial experience of RAP as a tummy bug and feeling very unwell. Interestingly, however, when asked why they think children experience tummy pains, while two mentioned diet, all five children made reference to worry, anxiety or stress. This perhaps conveyed the very different stage they were now at, having engaged with psychology services and likely gained a greater understanding and awareness of their pain.

### Theme 2: Contributing factors

It appeared that intrinsically linked internal and external factors were playing a role in the children’s RAP. There were many similarities in the internal factors that were revealed by the parents’ and children’s responses; e.g. anxiety and anticipatory worry. This finding is in line with the belief that childhood RAP is strongly associated with stress and anxiety (Rutten et al., 2015). Attachment-based factors also arose as a contributing factor for one child as they linked the pain to the absence of their mother. Although this was not explicitly identified among the other children, or indeed by parents, the potential role of attachment-based constructs may be an important factor in the onset/offset and maintenance of pain (Donnelly & Jaaniste, 2016).

External factors such as social situations, school and bullying were stressors clearly identified as contributing to significant bouts of RAP. Interestingly, the shift from in person schooling to home-schooling due to the COVID-19 pandemic brought the role of school as a stressor into focus, with some parents noting reductions in experienced RAP and increased engagement in school work during home-schooling. Equivalently, it was evident from child responses that negative social experiences and unpleasant environmental factors were triggers for their pain. Thus, these findings appear to be in agreement with the general consensus that emotional and environmental factors contribute to the manifestation of RAP (Newlove-Delgado et al., 2017). Some parents revealed other external factors such as family issues as likely contributing factors. This finding corresponds with the limited cross-sectional and qualitative research on the association between stressful and traumatic life events and RAP (Boey & Goh, 2001; Robinson et al., 1990). However, cross-sectional investigations have found no difference in levels of negative life events among patients with RAP compared with other patient groups (Walker et al., 2001).

### Theme 3: Coping mechanisms/pain management strategies

It was evident from both parental and child data that coping mechanisms and pain management strategies played a key role in managing the RAP. Interestingly, the strategies used seemed to change and develop over time, from basic in the initial stages to more advanced techniques as a greater awareness and familiarity with RAP was obtained. For example, parents cited drinking liquids, going to the bathroom and getting air as initial strategies. Over time, strategies developed more to encompass reassurance, effective communication to help find out potential causes and the use of distraction techniques. This may reflect a process of parents becoming more aware over time of the potential role of emotions in RAP. Indeed, a greater awareness and familiarity with RAP over time seemed to evoke a new and effective form of experiential reassurance amongst parents. This involved reminding the child of previous episodes of pain, how they got through it before and that they could do it again. These forms of pain management following the establishment of the absence of any underlying medical condition are consistent with the literature in this area (Brown et al., 2016; Huertas-Ceballos et al., 2008; Plunkett & Beattie, 2005).

Distraction and occupying ones-self appeared to be useful techniques for the children. Engaging in activities such as computer gaming and training were identified as pain-free experiences by some, suggesting that being in a state of flow has positive benefits (Nakamura & Csikszentmihalyi, 2009). Other children referenced consciously changing their thinking towards more positive thoughts, as well as breathing exercises and techniques learned through engaging with psychology as helpful approaches during episodes of pain. Consistent with parental experience, the role of psychology was found to be beneficial for children in that it generally enhanced their understanding and awareness of RAP and improved how they expressed themselves emotionally. Whilst the effects of psychological intervention have been found to be effective in treating children with RAP (Sprenger et al., 2011), only a limited number of small-sized studies are available for review. This highlights the importance of studies, such as this, that examine the child’s perspective in detail.

Having support from parents, other family members and friends also emerged as an important coping mechanism for children. It was evident that they found having someone to talk to about their experience of pain beneficial. Receiving reassurance appeared to form an integral component in this form of coping. This is in keeping with studies which highlight variables such as coping strategies, social support and cognitive processes (such as locus of control and self-efficacy) which are thought to facilitate psychological and functional adjustment in children with pain problems and contribute independently to adjustment (Kaminsky et al., 2006).

Resilience and determination also emerged as coping mechanisms from children’s narratives as they discussed getting on with various tasks and activities despite experiencing RAP. Interestingly a number of children displayed determination to not let the RAP stop them from doing things. Thus resilience and determination appear to build up over time through awareness and familiarity to RAP. Although, as these children had been engaging with a psychology service, it would also be reasonable to suggest that psycho-education had played a part in the growth of this resilience. Indeed, enhancing resilience has been recognised as one of the roles of psycho-education in dealing with RAP (Bufler et al., 2011; Sieberg et al., 2011). As well as furnishing coping strategies for dealing with acute pain and stress reduction, psychological intervention involving cognitive methods such as distraction techniques and cognitive restructuring may have changed how the child perceives their RAP, such that it is no longer an uncontrollable event, but instead a trigger for the use of pain coping strategies (Bufler et al., 2011).

### Theme 4: Impact and burden of RAP

A negative impact and burden of RAP was evident from both the parental and child data. RAP was shown to have a considerable emotional impact on the child both in terms of their reaction to the pain itself and due to the impeding nature of the experience. Notably, emotional reactions to RAP differed amongst the children, ranging from fear and upset to annoyance and frustration, and also changed over time. This is congruent with the interpretation outlined above, that how RAP is perceived, changes over time, potentially as a result of familiarity and awareness.

Sleep was also found to be affected by RAP resulting in poor functioning the following day, often impacting concentration, performance and attendance at school. The impact on sleep and school appeared intrinsically linked in terms of the burden it placed on the child, however experiencing RAP during school was found to generally make school more difficult and resulted in missed periods of class and having to catch up. These findings are synonymous with the existing literature which highlights the significant impact of RAP on school functioning (Chambers et al., 2004; Haim et al., 2004; Huntley et al., 2007; Ramchandani et al., 2007).

RAP was also found to be impeding everyday life for some children, as it stopped them from doing various tasks and activities. Interestingly, some parents saw this as more of an issue in the beginning, suggesting that over time the pains had less of an impact on the quality of life of their child. However, RAP was not a barrier all the time. Children mentioned not letting RAP stop them, but that it made things more difficult. Some parents felt that their child’s social life was not impacted by RAP. This was somewhat supported by the children, who stated they either did not or were less likely to experience RAP when with their friends. These findings support the claim that stressors and certain environments can act as contributors to RAP (Kakotrichi et al., 2016). It is also worth noting that the reduction in pain while with friends was not a universal experience, and that RAP did impede the activities and social engagement of some children, especially during particularly bad periods. Overall, RAP was found to have a significant effect on the quality of life of the child, which reflects existing literature in this area (Sjölund et al., 2020; Varni et al., 2015; Youssef et al., 2006).

A further noticeable trend was the impact that the child’s RAP had on the parents themselves and family quality of life in general, particularly in the earlier stages. Congruent with much of the previous literature (Bufler et al., 2011; Calvano & Warschburger, 2018), the initial uncertainty leading to an often drawn out and stressful process of seeking treatment and answers, as well as ruling out serious underlying medical conditions can have a serious negative impact on parents and families quality of life. Whilst it was found that parental worry was reduced following medical testing, for some the worries of something more serious persisted. It was clear that this combined with the on-going concerns about how the RAP was affecting their child was having a significant impact on parental quality of life overall, and this is consistent with the literature (Brodwall et al., 2018).

## Limitations

The findings of this study must be considered in light of a number of methodological issues. Although this study provided an enlightening insight into parents’ and children’s understanding and perceptions of RAP, and its impacts and burden, it was an exploratory study with a relatively small sample size. This was due to the impact of COVID-19 restrictions on recruitment. This limits the conclusions that can be drawn and the transferability of results beyond the participants of this study. A further limitation associated with the impact of COVID-19 was the uncontrolled setting for the interviews, as they had to take place online. Of the five child participants, four requested the presence of their parent in the room during their interview. Face-to-face interviews in the familiar hospital outpatient setting may have increased the possibility of children being interviewed alone. The presence of parents for the majority of interviews also raised the possibility that the presence of a parent may have influenced children’s responses. The non-validated nature of the interview could also be considered a limitation of this study; however, in the absence of a validated interview in line with the aims of this study, the interview questions were developed from a combination of those based on previous related research and in collaboration with an expert in the area.

The online aspect of the interviews may also have impacted recruitment, with parents and children possibly being reluctant to take part in an interview via video conference. There may have also been some benefits in the interview being conducted in this way, insofar as participants may have felt more comfortable and confident in the surroundings of their own home rather than in a clinical setting leading to more open responses and richer data. This concept is something that has been noted in other studies using online interview methods (Hanna, 2012; Lo Iacono et al., 2016), particularly for shy or introverted participants, who in a familiar environment might feel more comfortable opening up in front of a screen (Seitz, 2016).

## Strengths

Despite a small sample, a strength of the study was the recruitment of children of different ages (9, 10, 12 and 16) and sexes (three girls and two boys). This allowed for more range and diversity in responses and a greater insight. The sample had also all been experiencing RAP for more than a year, with the majority having a number of years’ experience. As a result, all the participants had been engaging with a psychology service and parents and children were able to provide an interesting perspective on RAP before and after psychological intervention. It could be argued that the inclusion of those at an earlier stage of their RAP journey may have provided different perspectives and this may have impacted interview responses. However, the initial stages of the RAP journey remained salient for many participants which enabled this study to provide insight across the journey, increasing the degree to which findings may be transferable. Future studies should attempt to recruit a broader sample to determine if these findings are systemic or not and to assess the commonality of these findings for a broader spectrum of the population.

A further strength of this study is that it is data driven. The inclusion of more primary data is an approach that allows participants to speak for themselves and provides a rich source of information for the reader that is distinct from the interpretations of the researcher (Morse et al., 2002; Wolcott, 1990). As such, a narrative logic was adopted, with the data presented in a way that tells a story, transitioning from one area to another in a sequence that best relates to the details of the story (Polkinghorne, 1995). As in any qualitative investigation, it is important to acknowledge that subjectivity and the researcher’s background could have influenced the design, collation of data and analytic processes. Strict adherence to rigorous quality guidelines and full transparency of the research process including collation of data and analysis were adopted in order to minimise researcher bias. Furthermore, the researcher’s position in this investigation was as an outsider who was new to this area and concept, and therefore would not have shared the experiences of the study participants.

The unique insight into both parents’ and children’s perspectives and understanding of RAP, along with its impacts and overall burden is perhaps this study’s main strength. To the researcher’s knowledge, no previous studies have examined both perspectives, providing a greater overall understanding of RAP. Moreover, this study is further strengthened by the rich and detailed accounts provided by many of the participants that encompass past and present experiences and perspectives. This provides a greater understanding of the RAP journey and how different aspects develop and change over time, including how psycho-education and psychological intervention can potentially influence this.

## Recommendations and conclusion

The findings of this study prompt a number of clinical recommendations. In relation to the uncertainty and worry of serious illness that parents expressed; it is important that during the period in which medical investigations are on-going, that parents are given information on the role of stress in physical health and how the presentation of stress in children can often manifest in more than one physical symptom.

It would be helpful to explore the internal factors identified in this study with parents from the earliest medical consultations. These conversations may often be initiated within general practice. If the role of sensitive temperament in children’s physical symptoms and the association between high levels of sensitivity and children’s experience of pain are explored at these early stages, it can facilitate timely referral to services such as primary care psychology or occupational therapy. Further, the fact that children highlighted anxiety specifically supports the need for on-going provision of anxiety prevention and interventions in school and in primary care settings.

The external factors mentioned by parents highlight the correlation between social anxiety and RAP (Cunningham et al., 2013; Dufton et al., 2009). The pathway for psychological intervention for children with social anxiety may be particularly challenging; as their difficulties will often not reach the threshold for child and adolescent mental health services and the long waiting times for other services may lead to exacerbation of the child's physical symptoms. The length of time children with RAP are waiting for intervention may also lead to more significant impact on their day-to-day functioning, such as increasing school absences.

The findings demonstrating the strategies that children use are also insightful. Given that these strategies might be difficult for children to use in a classroom setting, increased knowledge for teachers on their benefits would be helpful in ensuring that children with RAP are managing school as well as possible. Further, the benefit of awareness and knowledge of RAP found in this study, underlines the importance of a collaborative approach in health services, whereby medical professionals engage parents and children in the management of the child's symptoms.

The benefit of psychological support has been clearly demonstrated and highlights the importance of accessible services for children experiencing RAP. Indeed, children attending hospital services may not always have access to psychological supports within the hospital system, as access and referral criteria differ depending on resources. This study emphasises the importance of children and their parents engaging with a psychology service within a short time of the family receiving reassurance that there is no serious medical illness underlying the child’s symptoms.

In summary, study findings have important clinical implications in terms of increasing knowledge of the identification, treatment and management of children with RAP and subsequently enhancing clinical practice. They may also contribute to improving communication between clinicians, parents and children by providing insight from multiple perspectives into how RAP is experienced. Most significantly, this study makes an important contribution to the dearth of information concerning the experiences, journey of identification and impact of RAP, particularly for those living in the Republic of Ireland.

Exploratory analyses are frequently used to generate hypotheses for future research (Komorowski et al., 2016), and as such some of the current findings may inform future enquiry. Indeed, future research utilising larger samples would be beneficial. It is also important to note, that naturally many of the themes derived from this study were influenced by the families’ current, on-going psychological treatment. Therefore, it may also be worthwhile for future qualitative studies to focus on a psychology-naïve group of children and parents who have yet to engage with a psychology service. Such studies focusing on a different time point would likely develop insightful themes from the earlier stages of RAP and could provide a different perspective on the themes that emerged in this study. Particularly in their perceptions and understanding of RAP before psychology input and the factors that contribute. Research methods such as narrative enquiry would also enable the triangulation of data (Flick, 2018), and thus gain a more comprehensive understanding of RAP. Future quantitative research could aim to try and establish why some strategies work better for some children than others and the potential role of child or parent personality traits as a risk factor for RAP. Longitudinal studies could also be useful to track change in RAP over time and to see if there are any aspects of personality, environment or attachment relationships that predict this change.

In conclusion, this study has contributed to the existing literature on childhood RAP, and provided qualitative enquiry on parents’ and children’s perspectives on the topic. This study has further highlighted the overall impact and burden of RAP on the child and also on the parent and wider family. It has identified interesting changes that occur in the experience of RAP over time, through what appeared to be gaining a greater awareness and familiarity, as well as effective coping mechanisms and pain management strategies learned through experience and engagement with psychological services. This information can provide clinicians working with this population with an awareness of what these families may experience at different time points and stages of RAP, as well as the overall impact it can have on their lives. Consequently, there is potential for these findings to contribute to shaping psychoeducation and interventions for children experiencing RAP and informing professionals and clinicians in their approach.
